# Mediastinal Teratoma in a Twin Pregnancy: A Case Report

**DOI:** 10.7759/cureus.73074

**Published:** 2024-11-05

**Authors:** Joana Freire Gameiro, Helena Cunha e Carmo, Maria Palma, Raquel Ilgenfritz, Antónia Santos

**Affiliations:** 1 Prenatal Diagnostic Department, Unidade Local de Saúde Almada-Seixal, Almada, PRT; 2 Pathology Department, Unidade Local de Saúde Almada-Seixal, Almada, PRT

**Keywords:** fetal hydrops, fetal mediastinal teratoma, intrauterine fetal demise, mediastinal mass prenatal diagnosis, prenatal multidisciplinary management, twin pregnancy complications

## Abstract

Fetal mediastinal teratomas are rare tumors that can lead to serious complications such as fetal hydrops and intrauterine fetal death. Early prenatal diagnosis is critical in patient counseling, management, and preparation for postnatal interventions. In this report, we present the case of a 27-year-old woman in the second trimester of a dichorionic diamniotic twin pregnancy, in which the presenting fetus was diagnosed with a mediastinal teratoma and subsequently developed fetal hydrops, leading to intrauterine death. The diagnosis was initially made via ultrasound and the second twin exhibited no major anomalies. Following the fetal demise of the affected twin, a cesarean section was performed due to suspected maternal complications, and the surviving twin was delivered prematurely. This case highlights the importance of multidisciplinary collaboration in the management of complex twin pregnancies and underscores the challenges of diagnosing and managing rare fetal anomalies.

## Introduction

When evaluating the thorax, it is essential to be aware of how to approach fetal chest masses and consider the key questions that guide differential diagnosis, such as normal chest dimensions, deviation of the fetal heart axis, presence of the stomach, solid or cystic component, simple cyst or complex cystic mass, Doppler flow, location, extension beyond the chest wall and presence of hydrops [1].

Fetal teratomas are rare tumors, with an incidence of one in 40,000 live births [2]. A kind of extragonadal germ cell tumor, teratomas consist of any combination of disorganized tissues from the three germinal layers. While most commonly found in the sacrococcygeal area, approximately four percent occur in the mediastinum [3].

Ultrasonographic findings of a mediastinal teratoma typically show a complex heterogeneous mass containing both cystic and solid components, sometimes with calcifications. These masses are usually centrally located in the anterior mediastinum, pushing the lungs laterally. Pleural effusions may be present, giving a wing-like appearance on imaging. Color Doppler typically reveals variable vascularity without a dominant feeding vessel [1].

The differential diagnosis between mediastinal teratoma and lymphangioma is crucial due to their overlapping presentation but distinct characteristics. While teratomas often display a combination of cystic and solid components, lymphangiomas are typically septated, purely cystic masses without calcifications or significant solid areas. Lymphangiomas generally exhibit no Doppler flow and, although they may extend into the mediastinum, most of their mass is usually external to the chest, often extending into the neck or axillae. This cystic and avascular profile helps differentiate lymphangiomas from teratomas, which are more likely to have mixed tissue composition and internal vascularity on Doppler imaging [1,2].

About 80% of teratomas are benign, but their rapid growth and mass effect in the mediastinum can cause serious complications due to compression of vital structures like the great blood vessels, heart, esophagus, trachea, and lungs, potentially leading to fetal hydrops, fetal death, and respiratory distress after birth. The prognosis depends on the size and location of the mass and the presence of hydrops. The neonatal prognosis is associated with the degree of pulmonary hypoplasia and tracheomalacia [1-5]. In contrast, lymphangiomas, while potentially large, are less likely to cause such compressive effects [1].

Although in-utero resection has been reported [5], treatment mainly involves surgical resection post-delivery or ex-utero intrapartum treatment in cases where the fetal airways are compromised [1,3,6]. Prenatal diagnosis and early detection are crucial for appropriate counseling, management, and preparation of a multidisciplinary team for birth [4].

## Case presentation

**Figure 1:**
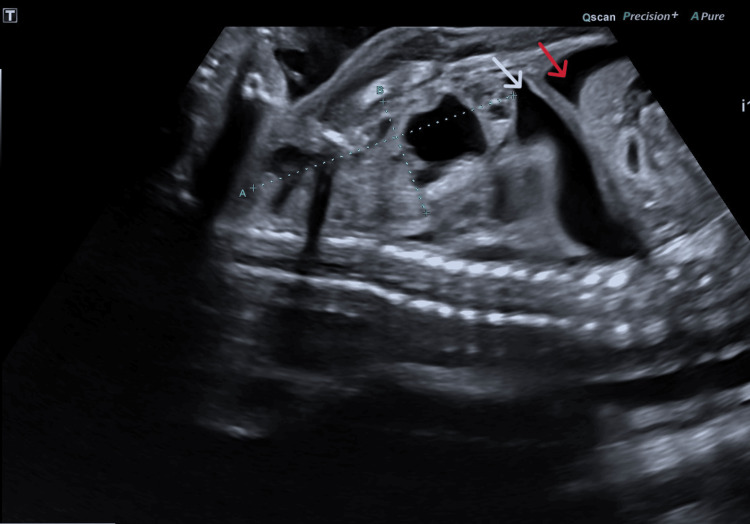
Sagittal ultrasound view of the fetal thorax and abdomen demonstrating mediastinal mass measuring 61 x 26 mm, pleural effusion and ascites. White arrow: pleural effusion; red arrow: ascites

**Figure 2:**
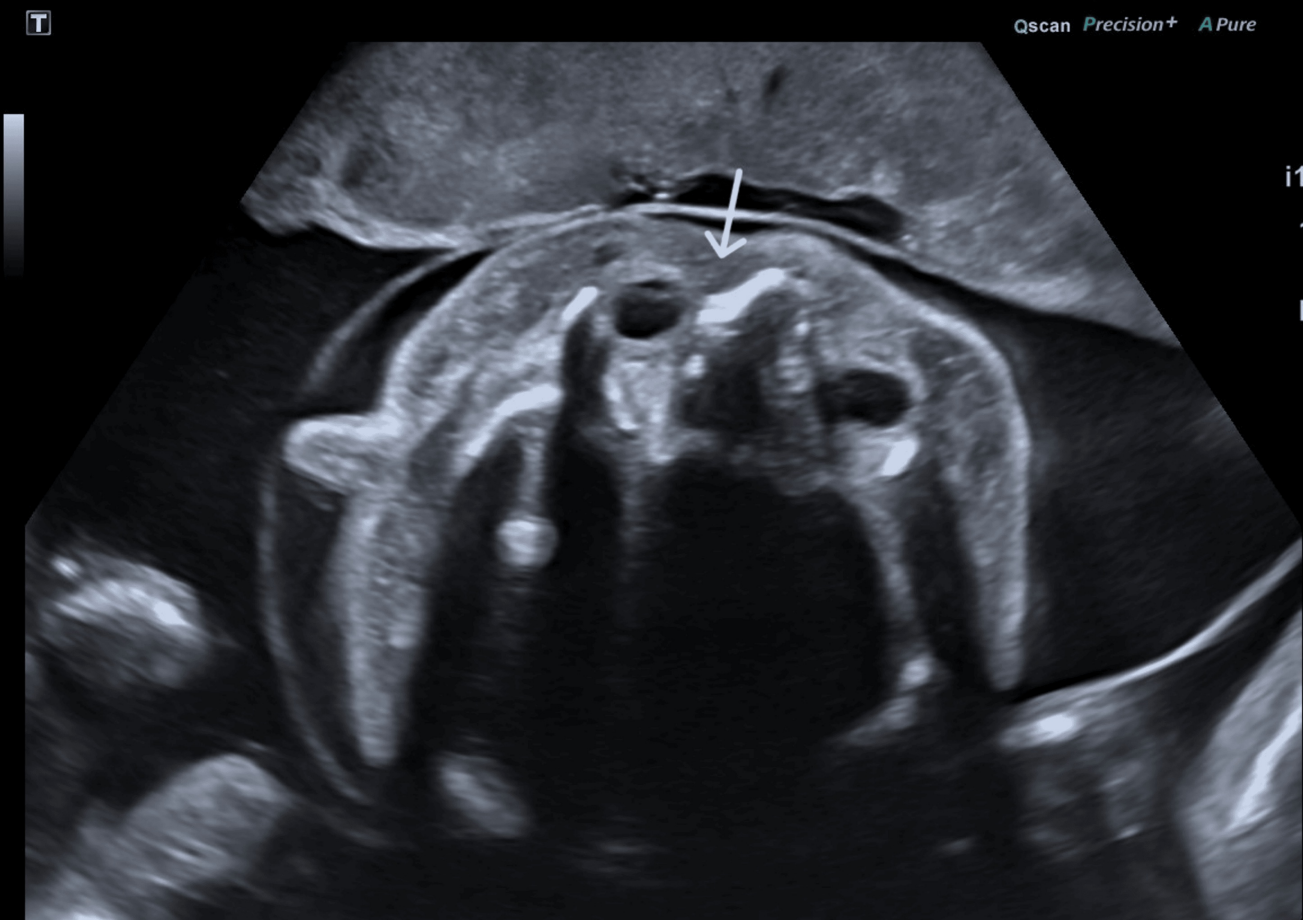
Transverse ultrasound view of the head demonstrating subcutaneous edema. White arrow: subcutaneous edema

**Figure 3:**
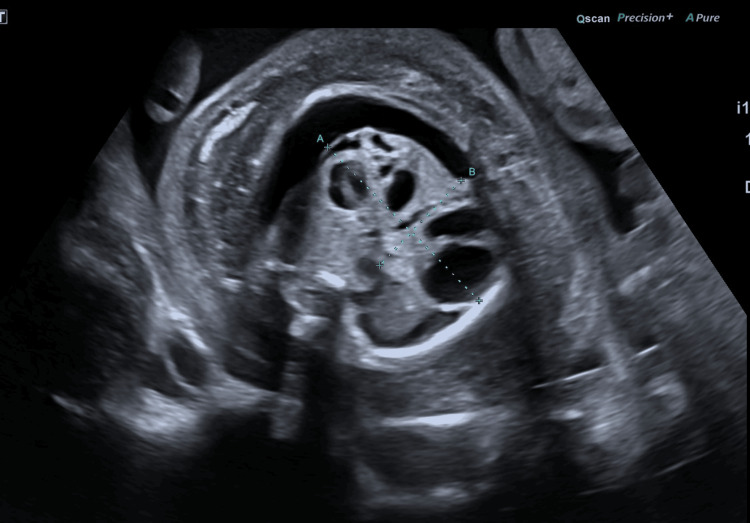
Transverse ultrasound view of the thorax demonstrating an irregular, multiseptated mass with a minor solid component, measuring 38.5 x 21 mm.

**Figure 4:**
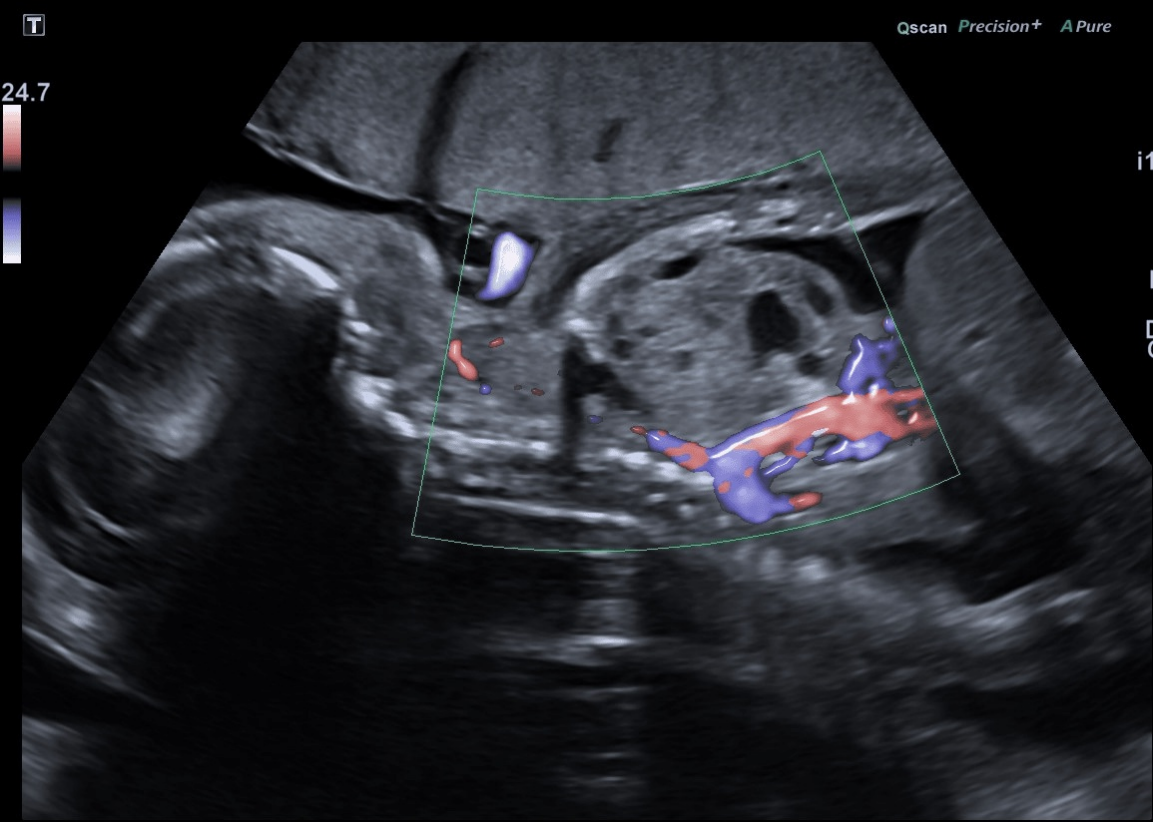
Sagittal ultrasound view of the fetal thorax and abdomen demonstrating the mediastinal mass with no color Doppler flow.

A 27-year-old woman, with three vaginal deliveries, uncomplicated and uneventful previous pregnancies, no history of congenital anomalies, and no past medical history, was referred to our hospital for follow-up on a spontaneous dichorionic diamniotic twin pregnancy. The 18-week ultrasound was normal and the patient missed the 20-22-week second-trimester anatomy scan because she was ill and was only able to reschedule it for the 24th week. The 24-week routine ultrasound revealed a mediastinal mass and fetal hydrops (Figures 1, 2) in the presenting fetus. The mass was described as irregular, multiseptated with a minor solid component, with no blood flow on Doppler, and measured 38.5 x 21 mm (Figures 3, 4). It was located in the mediastinum, extending to the fetal neck, and associated with pleural effusion and a wing-like appearance of the lungs (Video 1). The estimated fetal weight was 857 g, 81st percentile, with normal amniotic fluid and Doppler flow. The placenta was anterior high with no apparent abnormalities. The second twin showed mild right hydronephrosis with no other abnormalities, an estimated fetal weight of 704 g, 33rd percentile, and normal Doppler flow and amniotic fluid.

**Video 1:** Wing-like appearance of the lungs on fetal ultrasound.

STORCH infections and *Parvovirus B19 *were ruled out, and a fetal echocardiogram had no structural cardiopathy. Maternal blood tests were normal. Magnetic resonance imaging (MRI) suggested a cystic lymphangioma. Based on the ultrasound and MRI findings, a diagnosis of lymphangioma was proposed, and a multidisciplinary team discussed the case. The risks of amniocentesis and pleural effusion drainage were explained to the parents, who opted against intervention due to concerns about harming the healthy twin.

At 26 weeks, a follow-up ultrasound confirmed the intrauterine death of the affected fetus. The patient was monitored closely, and fetal lung maturation for the second twin was initiated. At 27 weeks, the estimated fetal weight of the surviving twin was 945 g, 23rd percentile. The maternal blood pressure profile tended to hypotension. Neuroprotection with magnesium sulfate was initiated for the benefit of the second twin. A suspicion of disseminated intravascular coagulation (DIC) was raised, after discussing the case with laboratory specialists, based on three consecutive clotted samples, a hemoglobin drop of approximately 2 g/dL in less than two days, a decrease in APTT from 23.9 seconds to 19.6 seconds (APTT control 28 seconds), a fibrinogen reduction from 597 mg/dL to 408 mg/dL, and a D-dimer level of 1.03 ug/mL.

After six hours of neuroprotection, a cesarean section was performed to save the mother and the second twin. The surviving twin was admitted to the intensive care unit and discharged after 66 days with sequelae related to prematurity. 

**Figure 5:**
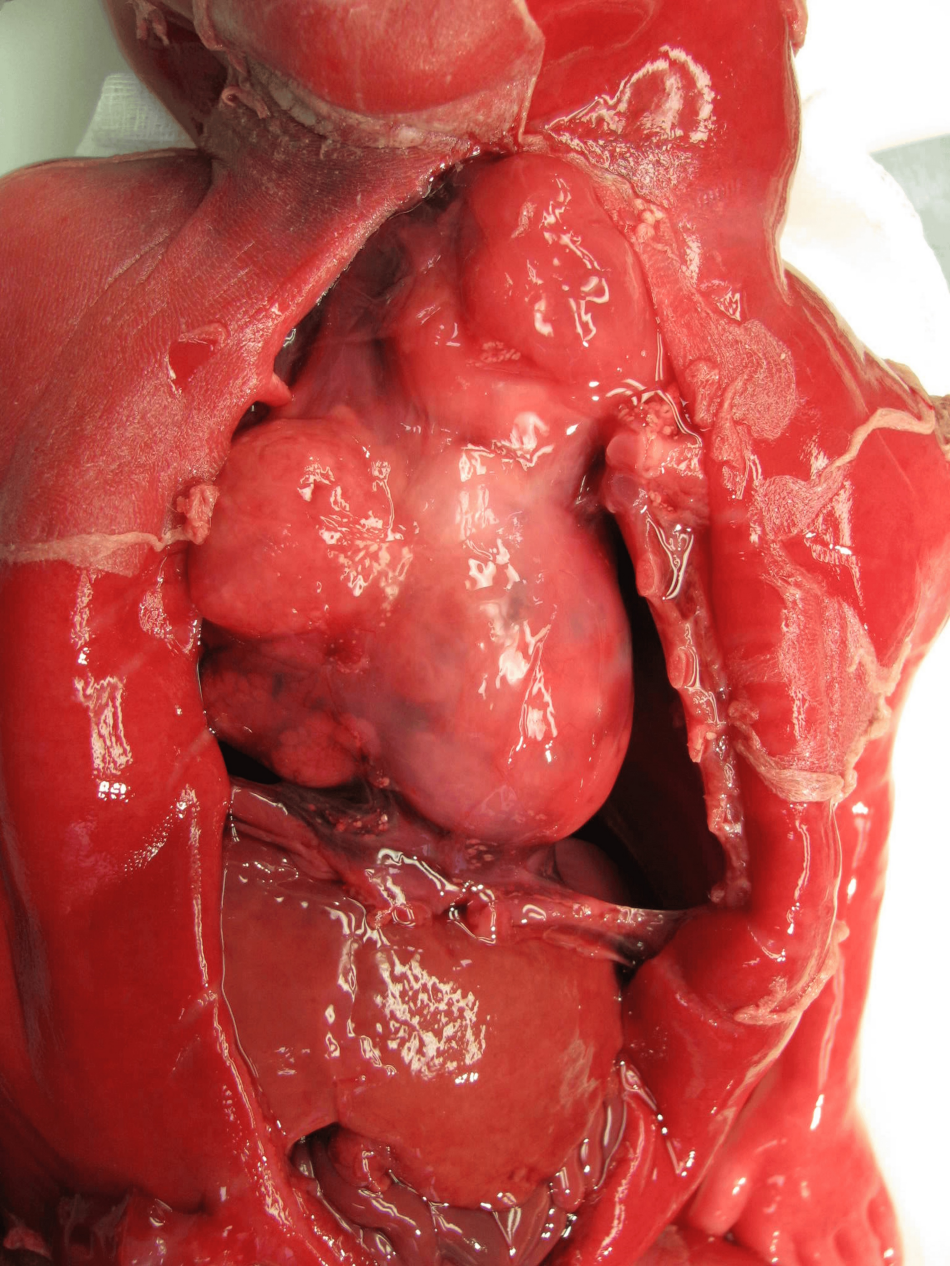
Fetal autopsy image demonstrating mediastinal teratoma in situ.

**Figure 6:**
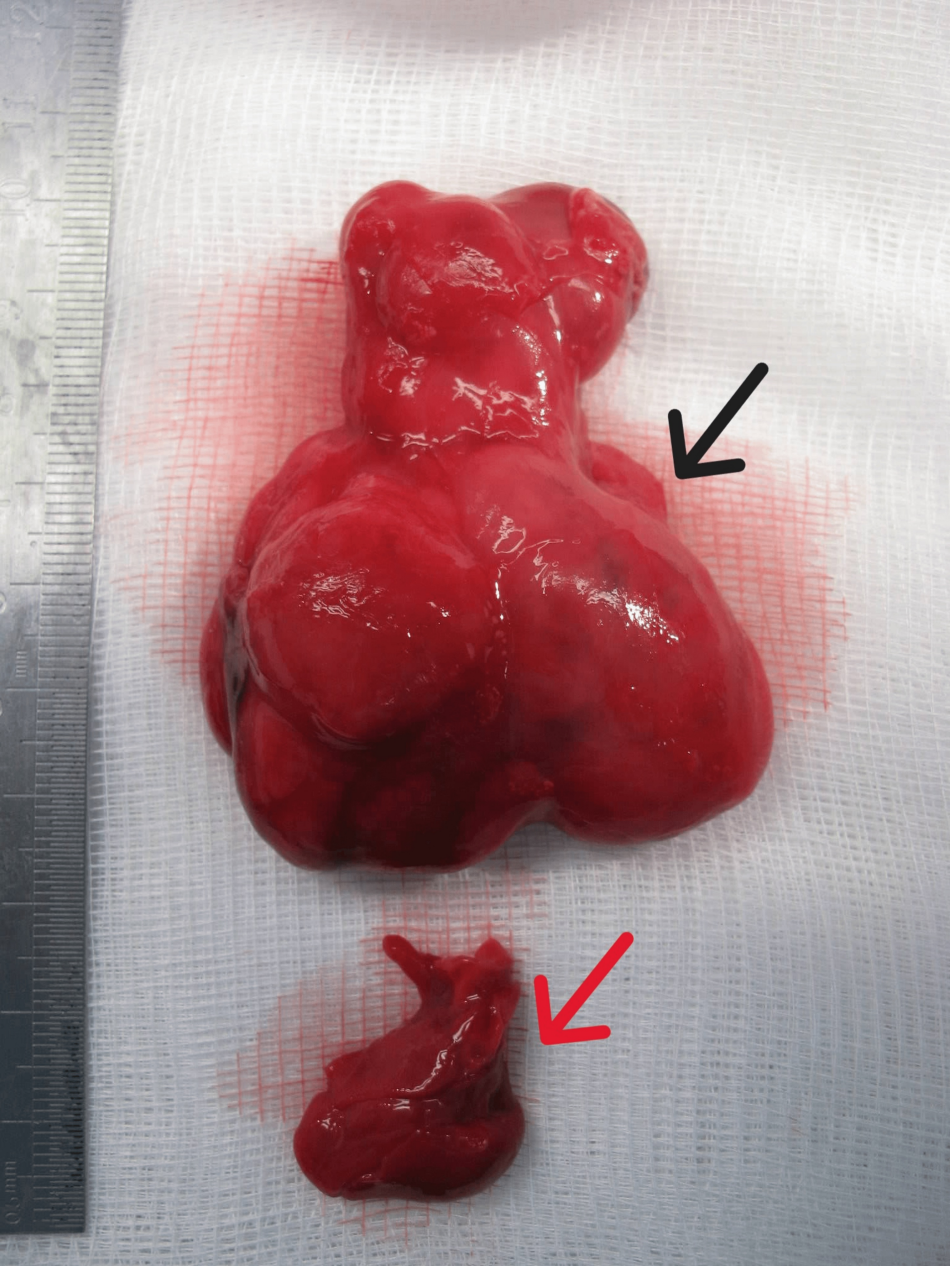
Fetal autopsy image comparing the mediastinal teratoma to the fetal heart. Black arrow: mediastinal teratoma; red arrow: fetal heart

The fetal autopsy report described the mass as cystic, involving the thymus and superior vena cava, with cells from all three germinal layers, including central nervous system tissue, cartilage, and respiratory epithelium, confirming the diagnosis of a trigeminal teratoma of the anterior mediastinum (Figures 5, 6). Asymmetric growth restriction was also noted.

## Discussion

This case illustrates a fetal teratoma in a twin pregnancy, complicated by the intrauterine fetal demise of the affected twin.

The differential diagnosis between teratoma and lymphangioma was challenging. Still, the presence of a minor solid component and the location of the mass in the mediastinum would support the diagnosis of teratoma, as opposed to lymphangioma. The latter usually shows most of its volume external to the chest in the neck or axillae [1]. Involvement of the superior vena cava justifies the severe hydrops and intrauterine fetal demise. The autopsy report was crucial to confirm the diagnosis.

Twin pregnancies have a higher risk for congenital anomalies, and although some are exclusive to multiple gestations, most are structural and affect only one twin. Therefore, the affected twin may pose a risk to the normal co-twin, hampering management and decision-making [6].

In this case, the diagnosis timing and the fact that the pregnancy was dichorionic were significant factors. Large masses associated with fetal hydrops are often fatal or linked to neonatal morbidity [1]. In some countries, such as Portugal, selective termination of pregnancy is an option up to 24 weeks + six days, which was a consideration in this case. However, selective termination carries risks, and in cases where an anomaly is lethal, the goal is to minimize the risk to the healthy twin. According to a study by Miremberg and colleagues [7], selective termination of the presenting twin resulted in a significantly higher rate of early complications such as preterm delivery, neonatal intensive care unit admission, and low birth weight. When a lethal anomaly threatens the co-twin's survival, prioritizing the healthy twin's health and safety is usually the best course of action [6]. In the case of intrauterine fetal demise in a dichorionic pregnancy, if maternal conditions potentially associated with fetal death are excluded, and the well-being of the survivor is established, no immediate intervention should be taken as these cases are not associated with fetal effects and the risk for the survivor is negligible [8]. Nonetheless, in twin pregnancies discordant for major fetal anomalies the unaffected fetus is at increased risk of preterm delivery and higher perinatal mortality [6].

According to Ong and colleagues, a systematic review [9], the risk of dichorionic co-twin death, neurological sequelae, and preterm delivery was 4%, 18%, and 57%, respectively. Fetal demise in the late second and early third trimester presents clinicians with the most difficult choices and the potential dilemma of either delivery of a premature twin or conservative management with the risk of morbidity and mortality of the survivor [8].

In this clinical case, the teratoma led to the fetal death of one of the twins, which, in turn, increased the risk of DIC, as was observed in this instance. DIC arises from the release of tissue factor after a trigger that leads to diffuse activation of the coagulation cascade culminating in a consumptive coagulopathy [10]. Unlike intrauterine fetal demise in singletons, maternal DIC is, for unclear reasons, extremely rare in twin pregnancies [8,10]. Nonetheless, the suspicion of DIC in the presented case demanded urgent cesarean delivery to protect both the mother and the healthy twin.

After 10 months of follow-up, the surviving twin appears to have multiple prematurity sequelae, including mild neurodevelopmental delay.

## Conclusions

This case demonstrates the complexity of decision-making in twin pregnancies where a severe anomaly is diagnosed late in one of the fetuses, such as a mediastinal teratoma. Early diagnosis in such cases allows for selective termination when desired by the parents, which can prevent extreme prematurity and improve the prognosis for the healthy twin. In this instance, the late diagnosis led to a more reserved outcome for the surviving twin. The prenatal diagnosis based on imaging was crucial for guiding initial clinical decisions, although the definitive diagnosis was only possible postnatally through fetal autopsy. This highlights the importance of continuous monitoring and the preparedness to adapt the management plan as more information becomes available after birth.

The demise of the affected twin, compounded by the risk factors introduced in a twin pregnancy, demanded careful consideration of the timing of delivery to protect the healthy twin. This case illustrates the need for a multidisciplinary approach and highlights the challenges in decision-making when prenatal diagnostics are limited and must be confirmed postnatally. The integration of all available diagnostic tools, including postnatal pathology, is essential in providing a comprehensive understanding of the case and guiding future management strategies in similar scenarios.
